# Remedies and Their Uses

**Published:** 1906-07-07

**Authors:** 


### REMEDIES AND THEIR USES.

### A Note on the Treatment of Cirrhosis of the Liver.

THERE is no drug known which has any effect on fibrous tissue formed in alcoholic cirrhosis of the liver. Iodide of potassium will cause gummata to disappear, but its effect on surrounding fibrous tissue is doubtful, and when the fibrous tissue is uniformly distributed throughout the liver as in alcoholic cirrhosis it is quite useless to give it. Many other drugs have been tried, but with no avail. Within the last few years thiosinamine has been given, by hypodermic injection, in cases where fibrous tissue has been developed, and sometimes with marvellous results. It was first given in cases of Duputyren's contracture—a disease in which surgical measures are often disappointing. In many cases reported the injections have caused a disappearance of the bands of fibrous tissue binding down the fingers and a restoration of the normal condition. In these cases massage was daily practised during the continuance of the injections. But massage is not a necessary adjunct to the action of thiosinamine, for a case has been reported of cicatricial contracture of the œsophagus, from swallowing a corrosive liquid, where the narrowing was so extreme that not even the finest bougie could be passed, and gastrostomy was contemplated, and where injections brought about a renewal of the power of swallowing both solids and liquids and a widening of the calibre of the œsophagus. These results suggested a trial of this drug in cirrhosis of the liver when a suitable case should turn up. At length a woman, aged thirty-four, was admitted suffering from undoubted alcoholic cirrhosis of the liver. She had been under observation two years previously with an enlarged liver, extending three inches below the costal margin, and occasional hæmatemesis, and had gone out unimproved. Three months ago she was again admitted suffering from the same symptoms, and with the liver in exactly the same condition. The only change was that the spleen was now slightly enlarged, extending one and a half inches below the costal margin. There was no jaundice and no ascites, and the patient was in very fair condition—not wasted or cachetic. The hæmatemesis was small in amount, half to one ounce was vomited once or twice a week. The treatment adopted was the injection of thiosinamine every other day. This was continued for two months. Not the slightest change was observed either in the size of the liver and spleen or in the occurrence of hæmatemesis. The patient increased a little in weight—owing, no doubt, to good food and to being kept in bed—and said she felt better. The treatment, then, was a failure. It is recorded that hopes may not be placed on it in other cases of the same disease. It might, perhaps, be added that a year or so ago thiosinamine was given a good trial in a case of mitral stenosis of some years' duration, but with no results. In that case, however, it was possible that calcification of inflammatory tissue might have taken place, and that the drug had not "had a fair chance."

It would seem, therefore, that thiosinamine has a limited sphere of action; though the cause of this is doubtful—possibly different causes bring about the formation of different kinds of fibrous tissue. A word may be added as to the technique of the injections. $1\frac{1}{2}$ gr. were dissolved in 15 ♏ of a mixture of water 80 parts and glycerine 20 parts with the addition of sodium salicylate ($\frac{1}{2}$ molecule of salicylate to 1 molecule of thiosinamine), and this was injected every other day under the skin in the loin or flank. Thiosinamine can be dissolved in these proportions of water and glycerine, but soon separates out in crystals if the sodium salicylate is not added. The injections do not cause pain or any induration or other trouble.

### Formalin in Ringworm.

FORMALIN is undoubtedly a very powerful germicide and a very weak solution reaching ringworm spores would rapidly kill them. The potency of this antiseptic may sometimes be accidentally illustrated by such a circumstance as the following. A culture of micro-organisms was killed by a small quantity of a 10-per-cent. solution of formalin, and the dead culture was thoughtlessly placed by the laboratory assistant in the incubator in which were living growths of diphtheria, typhoid bacilli, and other micro-organisms. The vapour from the formalin solution killed all the living cultures. If the vapour is so powerful, obviously a solution coming in contact with micro-organisms must be still more powerful; but in the case of ringworm the difficulty is to bring it into contact with the spores, since their deep situation in so many instances efficiently protects them from the action of all remedies. Those who have had experience of the use of formalin in this disease seem to think, however, that results show that it has greater penetrating power than the majority of applications used for ringworm. Mr. Mellish, who has recently written in THE HOSPITAL on the subject, has used strong formalin, that is, the 50-per-cent. solution of formaldehyde, with success. There is one objection to the use of strong formalin and that is the pain it occasions. Possibly this objection to its use might, at least in part, be removed by first applying a solution of cocaine. However that may be, solutions of formalin sufficiently weak not to give rise, at the most, to more than smarting of very brief duration, if used thoroughly, generally succeed in quickly curing the disease. Formalin ♏v. to methylated spirit ʒj., for example, is a solution of sufficient strength to be efficacious without causing pain. A solution in spirit is better than one in water because the spirit is, in part, a solvent of any oily matter that may be present, and should it be thought desirable to also apply an ointment, the rapid evaporation of the spirit allows the ointment to be rubbed in quickly after the application of the lotion. In the event of the weak solution of formalin failing to effect a cure, strong formalin no doubt may be tried before the question of the $x$-ray treatment is considered. At least one case of ringworm which had been treated unsuccessfully by a preparation of formalin was afterwards quickly cured by the $x$-rays.

